# Draft Genome Sequence of *Vibrio parahaemolyticus* Strain M0605, Which Causes Severe Mortalities of Shrimps in Mexico

**DOI:** 10.1128/genomeA.00055-14

**Published:** 2014-03-06

**Authors:** Bruno Gomez-Gil, Sonia Soto-Rodríguez, Rodolfo Lozano, Miguel Betancourt-Lozano

**Affiliations:** CIAD, AC Mazatlan Unit for Aquaculture and Environmental Management, AP 711 Mazatlán, Sinaloa, México

## Abstract

Acute hepatopancreatic necrosis disease (AHPND), also known as early mortality syndrome (EMS), causes high mortalities in cultured shrimps in Asia (L. Tran et al., Dis. Aquat. Organ. 105:45–55, 2013, http://dx.doi.org/10.3354/dao02621). Here, we report the draft genome sequence of one Mexican strain of *Vibrio parahaemolyticus* that causes similar clinical signs in diseased shrimps.

## GENOME ANNOUNCEMENT

Several strains were isolated from diseased cultured shrimps in Sinaloa, Mexico. They were identified as *Vibrio parahaemolyticus*, and laboratory challenges proved that several of them are able to cause severe mortalities in shrimps (unpublished data). One strain, *V. parahaemolyticus* M0605, was isolated from the stomach of *Litopenaeus vannamei* cultured on CHROMagar Vibrio as a mauve-colored colony in Eldorado, Sinaloa, Mexico, on 22 July 2013.

DNA was extracted with a Promega kit and sequenced with an Ion Torrent platform at CIAD Mazatlán. We obtained 403,443 reads (average, 179 bp), for a total of 158.4 Mbp. One hundred fifty-seven contigs (>100 bp) were assembled with Geneious version 7.0.5 (*N*_50_, 126.2 Kbp), for a genome size of 5.650 Mbp (20.0× coverage). The contigs were annotated in RAST (1); 5,152 coding sequences (CDSs) and 128 RNAs were found. Chromosome scaffolding and synteny were obtained as described earlier (2).

M0605 has two chromosomes (Ch) of approximately 3.356 Mbp for ChI (3,145 CDSs) and 1.767 Mbp for ChII (1,609 CDSs). Nine prophages were located in the two chromosomes, with five in ChI and four in ChII, ranging from 5.9 to 58.2 Kbp. Prophage f237 was found on both chromosomes. An 89.5-Kbp integron was detected in ChI, with approximately 115 CDSs in ~95 gene cassettes.

Four plasmids were detected: two IncP plasmids of 95.4 Kbp (45.6% G+C, 113 CDSs) and 50.6 Kbp (46.3% G+C, 54 CDSs), an IncF plasmid of 54.1 Kbp (44.0% G+C, 57 CDSs), and another IncP 40.6-Kbp plasmid (40.8% G+C, 40 CDSs) inserted in chromosome I.

Several pathogenicity mechanisms were identified on both chromosomes: five iron acquisition systems (hemin, enterobactin, vibrioferrin, and two TonB) and seven secretion systems (two type 2 secretion systems [T2SS], one T3SS, two T2/4SS, and two T6SS). At least 14 different toxin genes were annotated, two of which are large repeats in toxin (RTX), as well as nine hemolysins. Several proteases were found, three of which are zinc-dependent proteases, as well as one vibriolysin; five chitinases and a Tfox chitin metabolism regulator were also detected. One adherence system (type IV pilus) and one antiphagocytosis (capsular polysaccharide) system were identified. Two quorum-sensing systems are present, LuxPQ and LuxMN.

### Nucleotide sequence accession numbers.

This whole-genome shotgun project has been deposited at DDBJ/EMBL/GenBank under the accession no. JALL00000000. The version described in this paper is version JALL01000000.
